# Bacterial Profiles of Brain in Downer Cattle with Unknown Etiology

**DOI:** 10.3390/microorganisms11010098

**Published:** 2022-12-30

**Authors:** Yeong-Jun Park, Gi-Ung Kang, Minsoo Jeong, Vineet Singh, Jongho Kim, Kyunghyun Lee, Eun-Jin Choi, Heui-Jin Kim, Seungjun Lee, Sook-Young Lee, Jae-Ku Oem, Jae-Ho Shin

**Affiliations:** 1 Department of Applied Biosciences, Kyungpook National University, Daegu 41566, Republic of Korea; 2 Animal and Plant Quarantine Agency, Kimcheon-si 39660, Republic of Korea; 3 College of Veterinary Medicine, Jeonbuk National University, Iksan-si 54596, Republic of Korea; 4 Department of Food Science and Nutrition, Pukyong National University, Busan 48513, Republic of Korea

**Keywords:** downer cow, unknown etiology, brain microbiota, next-generation sequencing

## Abstract

Downer cow can be caused by muscular paralysis, neurological damage, metabolic disorder, and/or the complication of microbial infection. However, downer cow with unknown etiology is issued because of the non-detection of its bacterial etiological agent. In this study, differences in the bacterial community in brain tissues between downer cattle with unknown etiology and healthy slaughtered cattle are investigated. Bacterial diversity and representative genera between downer and normal cattle were significantly different (*p* < 0.05). There are significant differences in representative genera of downer and normal cattle, especially the significance, fold change, and area under the receiver operating characteristic curve score (*p* < 0.05). Furthermore, the prediction of functional genes in brain microbiota between the downer and normal cattle revealed differences in the cluster of orthologous gene categories, such as lipid transport and metabolism, secondary metabolite biosynthesis, and signal transduction (*p* < 0.05). This study revealed a significant difference in microbiota between the downer and normal cattle. Thus, we demonstrate that representative genera from downer cattle through 16S rRNA gene amplicon sequencing and microbiota analysis have the potential as candidates for bacterial etiological agents for downer cow.

## 1. Introduction

Cattle occupy a significant portion of the livestock industry and have been important in agriculture. Especially, downer cow (DC) is a clinical sign wherein the cattle enter a non-ambulatory state [1,2]. The DC occurred for several reasons as follows (1) the metabolic disorder caused by minerals; (2) paralysis by nerve and muscle damage after calving; (3) nervous and musculoskeletal problems; or (4) systemic disease caused by the toxic material, metabolic disorder, or neurological and alimentary conditions [1,2]. In addition, DC can be caused as a clinical sign of infection with encephalitis, meningitis, or meningoencephalitis by *Haemophilus* and *Listeria* genera; and botulism caused by *Clostridium* genus [2,3]. However, in some cases of DC, the etiology remains unknown, evading identification in autopsy, clinical, or pathological investigation. According to a diagnosis report from the animal and plant quarantine agency of South Korea, downer cows without etiological agent are detected [4].

Microbiota analysis using the next-generation sequencing (NGS) technique provides insight into the relationship between a disease and microorganisms [5,6]. Although fewer relevant studies have been conducted on the livestock industry than those on human disease, they contain valuable contributions to studying livestock disease and microbiota [7]. Previous studies investigated etiological agents, which can cause livestock diseases, such as bacteria, fungi, and viruses. Two previous studies have investigated the correlation between etiological candidates of non-suppurative encephalitis and abortion through viromes and microbiomes using whole metagenome and amplicon sequencing, respectively [8,9]. From these studies, using brain tissue and abomasal contents, candidate viruses and bacteria were identified and suggested as etiological agents for non-suppurative encephalitis and abortion, respectively. Thus, NGS and bioinformatic analysis can be considered as a potential tool not only to explore the correlation between diseases and microorganisms but also to find candidate causative agents of diseases.

The blood–brain barrier (BBB) is a selective semipermeable border related to molecule transport. Previous research revealed that the BBB prevents microorganisms from entering the brain and cerebrospinal fluid (CSF) [10,11]; however, bacteria, fungi, and viruses have also been revealed to disrupt the BBB and invade the brain and CSF potentially. Furthermore, these invasions may cause microbial infection in the central nervous system and neurological disease [12,13,14,15]. A recent microbiota study used CSF samples to investigate a neurological disease [16]. However, these kinds of studies also deal with animals in the field as well. 

In this study, the microbiota of brain tissue from cattle with DC having unknown etiology and normal cattle were compared. Additionally, representative genera that show differences between downer and normal cattle were investigated to find bacterial etiological agent candidates to aid the diagnosis of cattle with DC.

## 2. Materials and Methods

### 2.1. Bovine Brain Sample Collection and Diagnosis Based on the Necropsy, Pathological, and Clinical Examination

Brain samples from dead bovine that died of DC with neurological clinical signs such as depression, restlessness, ataxia, and astasia, were submitted to the Animal and Plant Quarantine Agency (Kimcheon-si, Gyeongsangbuk-do, Republic of Korea). To diagnose downer cattle, pathological diagnosis, clinical symptoms, mineral concentration such as calcium and magnesium, pathogenic bacteria isolation, virus detection, antibody test, and post-mortal change were checked [17]. In detail, pathological diagnosis, clinical symptoms, and post-mortal changes were decided by the veterinarian and veterinary pathologist of at least three people and summarized the results. Histopathological diagnosis caused by infectious agents was confirmed on the findings of lesions related with encephalitis (perivascular cuffing and gliosis) or meningitis (infiltration of inflammatory cells in leptomeninges). Calcium and magnesium concentrations in bovine blood were measured through an AU480 automated chemistry analyzer (Beckman Coulter, Brea, CA, USA). MacConkey agar and 5% sheep blood agar were used to incubate pathogenic bacteria such as *Histophilus somni*, *Listeria monocytogenes* in brain tissue. Identification of isolated bacteria was proceeded through VITEK^®®^ 2 Compact and VITEK^®®^ MS (BioMérieux, Marcy-l’Etoile, France) according to the manufacturer’s instruction. DNA and RNA samples from brain tissue were extracted for virus detection through the Maxwell RSC instrument, RSC Blood DNA kit (Promega, Madison, WI, USA), and RSC Viral TNA (Promega) according to the manufacturer’s recommendations. To detect viruses such as bovine viral diarrhea virus (BVDV), bovine herpesvirus-1 (IBR), akabane virus, Aino virus, bovine ephemeral fever virus, Chuzan virus, and Ibaraki virus, PCR was performed through LiliF IBR PCR kit, LiliF BD-Multi RT-PCR Kit (iNtRON Biotechnology, Seongnam, Republic of Korea), and VDx Single RT-PCR kit (MEDIAN Diagnostics, Chuncheon, Republic of Korea) according to the kit manual. Antibody test was performed to detect BVDV and Infectious Bovine Rhinotracheitis virus by Bovine viral diarrhea virus antibody test kit (IDEXX Laboratories, Inc., Liebefeld-Bern, Switzerland), Infectious Bovine Rhinotracheitis Antibody test screening format (Svanova Biotech AB, Uppsala, Sweden).

Before starting to conduct a necropsy, equipment such as a necropsy station, forceps, operating scissors, and scalpel were first washed using detergent and subsequently soaked into 1% Virkon S solution (Lanxess, Cologne, Germany) for at least over 10 min. Then, all instruments were sterilized at 121 °C for 15 min and dried in the oven. Necropsy of dead bovine was performed based on the necropsy manual, and brain tissues were sampled via sterilized forceps and scissors [18]. Normal cattle samples were secured immediately after being slaughtered. In addition, diagnosis and collection of brain tissue were performed within a day and stored in a −70 °C deep freezer.

Based on the diagnosis result, brain tissues of downer cattle were classified if there are any lesions, bacterial, and viral etiological agents were detected. Normal cattle heads were purchased from the slaughterhouse, and their diagnosis proceeded in the same way as that of downer cattle. Then, brain tissues with or without clinical signs were collected as brain tissues for the downer and normal cattle groups, respectively. Various brain tissue samples such as the forebrain, midbrain, cerebellum, hindbrain, and brain stem were collected (Table S1). All bovine brain tissues were classified according to their characteristics (Table 1). In 99 bovine brain tissues, 57 brain tissues came from downer cattle. The average age of downer cattle was calculated as 52 months and was higher than normal cattle. In the sex of samples, most of the downer and normal cattle were confirmed as female and male, respectively.

### 2.2. DNA Extraction from Brain Tissues

The tissues were rinsed through 70% ethanol and sterilized with distilled water thrice to remove microbial contamination. To prevent DNA contamination during microbiota analysis, a contamination test was conducted before starting to extract total DNA from the brain tissue [19]. In the contamination test, 70% ethanol waste and SDW waste from the brain tissue rinsing step was spread on a 1/10× BHI agar plate and incubated to check whether any bacteria remained or not. In addition, to check the presence or absence of bacteria in all reagents of the DNA extraction kit, all reagents were used as templates for 16S rRNA gene PCR. The total DNA extraction from the bovine brain tissue proceeded. Bovine brain tissues (0.1–0.2 g) were chopped and grounded using Biomasher II^®®^ Closed System Disposable Tissue Homogenizer (Kimble Chase, Tennessee, TN, USA) under the 180-μL ATL buffer and 20-μL proteinase K solution. QIAamp DNA mini kit (Qiagen, Hilden, Germany) was used for DNA extraction according to the kit manual for tissue and Gram-positive bacteria. The extracted DNA concentration was measured using a Qubit 2.0 fluorometer (Thermo Fisher Scientific, Waltham, MA, USA). The DNA samples were kept at −20 °C.

### 2.3. Sequencing Library Preparation and Amplicon Sequencing

All equipment in the library preparation, clonal generation, and sequencing via the Ion Torrent platform was used in the NGS Core Facility at the Kyungpook National University (Daegu, Republic of Korea). The sequencing libraries were produced by polymerase chain reaction (PCR) amplifying the variable region 4 to 5 (V4–V5) of bacterial 16S rRNA gene with two primers (515F; 5′-GTG CCA GCM GCC GCG GTA A-3′, 907R; 5′-CCG YCA ATT CMT TTR AGT TT-3′). PCR was conducted using EmeraldAmp PCR master mix (Takara, Tokyo, Japan) in 50-μL scale and based on the condition as follows: 3 min of initial denaturation at 95 °C, followed 1st PCR by ten cycles of denaturation (95 °C for 30 s), annealing (57 °C for 30 s), extension (72 °C for 30 s), followed 2nd PCR by 25 cycles of denaturation (95 °C for 30 s), annealing and extension (72 °C for one min), and a final extension at 72 °C for five min. The sequencing library concentration was measured via a Qubit 2.0 fluorometer (Thermo Fisher Scientific, Waltham, MA, USA) and pooled into a single pooled library. The sequencing library was validated using Agilent Bioanalyzer 2100 system (Agilent Technologies, Santa Clara, CA, USA). Emulsion PCR through Ion Torrent OneTouch was conducted and enriched a template-positive ISP clonal using Dynabeads MyOne Streptavidin C1 bead (Invitrogen, Waltham, MA, USA) according to the Ion PGM Hi-Q View OT2 400 kit manual. Ion Torrent PGM machine sequencing was conducted to analyze the sequence for 1250 flows based on the manufacturer’s instruction (Thermo Fisher Scientific, Waltham, MA, USA).

### 2.4. Pre-Processing of Sequence Data and Microbiota Analysis

Microbiota analysis with sequence data was performed using a data analysis server in the NGS Core Facility at Kyungpook National University (Daegu, Republic of Korea). The FastQC version 0.11.9 program checked the raw sequencing data quality [20]. Removal of chimeric sequence and construction of amplicon sequence variant (ASV) was conducted using QIIME 2 version 2020.08 pipeline, including DADA2 version 2020.08 software [21]. Using DADA2 software, sequences that show under Q30 value were trimmed, and taxonomic assignment of the ASV table was proceeded using sequences over Q30 value by trained classifier using Naïve Bayes training method and SILVA 138 99% database [22]. The ASVs, which presented a low frequency and were assigned as mitochondrial and chloroplast sequences in the abundance table, were eliminated. All samples in the ASVs table were rarefied and normalized at a minimal sequencing depth of 3911 reads.

For analyzing bacterial microbiota, including community structure, alpha diversity, and beta diversity, Calypso 8.4 version program, Phyloseq package version 1.30, and MicrobiomeSeq package version 0.1 in R software version 4.0.2 were utilized [23,24,25]. Briefly, the community structure was displayed using the top 20 abundant genera via a pie chart. Alpha diversity indices, such as Shannon and Richness index and beta diversity based on the Bray–Curtis and unweighted UniFrac methods, were calculated at the genus level. Representative genera were observed between experimental groups through feature selection using random forest regression, calculation of differential abundance, significance, fold change, prevalence, and area under the receiver operating characteristic curve (AUC) score. Random forest regression was used to determine the representative genus, which can classify downer and normal cattle through MicrobiomeSeq package version 0.1 in R software ver. 4.0.2. Representative genera selected through random forest regression confirmed their differential abundance, significance, fold change, prevalence, and AUC score through the check association function of SIAMCAT package version 1.6.0 in R software ver. 4.0.2 [26]. The calculation of AUC score, sensitivity, specificity, and receiver operating characteristic (ROC) curve visualization was conducted through pROC package ver. 1.17.0.1 [27]. The bacterial network of downer and normal cattle was also analyzed using the MicrobiomeSeq package version 0.1 in R software. The genus was determined based on the ASV table and SILVA taxonomy. The statistically significant genus was used and calculated using Spearman’s correlation. All genera displayed in the bacterial network showed greater than 0.5 of Spearman’s rho value and under 0.05 of *p*-value and *q*-value. The functional gene prediction based on the brain microbiota from the downer and normal cattle was conducted through the PICRUSt2 version 2.1.4 program [28]. In functional gene analysis, the 16S rRNA gene copy numbers of all ASVs were normalized. Functional genes were predicted based on the cluster of orthologous (COG) database. All COG IDs from the PICRUSt2 result were classified according to the COG category. STAMP ver. 2.1.3 program was used for data statistics and visualization [29]. In the data, statistically significant COG categories were displayed.

### 2.5. Statistical Analysis

In brain microbiota data from the downer and normal cattle, all data from sequencing were normalized based on the total sum scaling method to obtain the rarefied operational taxonomic unit table. Statistical analyses of alpha diversity, representative genus, and beta diversity were calculated using the Wilcoxon and PERMANOVA, respectively. In network analysis, Spearman’s correlation proceeded at the genus level and was shown to be statistically significant. In functional gene analysis, the Shapiro–Wilk test and Welch’s t-test were conducted. ANOVA was used for the multiple-group comparison test, and its correction was performed using the Tukey HSD test. Statistical significance was considered based on the *p*-value, which was less than 0.05.

## 3. Results

### 3.1. Diagnosis Result of Downer and Normal Cattle

To decide downer and normal cattle, diagnosis through pathological diagnosis, clinical symptom, mineral concentration such as calcium and magnesium, bacteria isolation, virus detection, antibody test, and post-mortal change was performed (Table S2). All samples in this result did not show any post-mortal change. As a diagnosis result of downer cattle, pathogenic bacteria, virus, or lesion in brain tissue was confirmed. Before starting the experiment, a cross-contamination test was performed. No bacteria are found on the BHI agar plate in the third waste of 70% ethanol and sterilized distilled water final washing waste. Furthermore, there is no DNA band in lysis, washing, elution buffer, and library preparation agent (Figure S1).

### 3.2. Differences in Bacterial Structure and Diversity between the Downer and Normal Cattle

**Figure 1:**
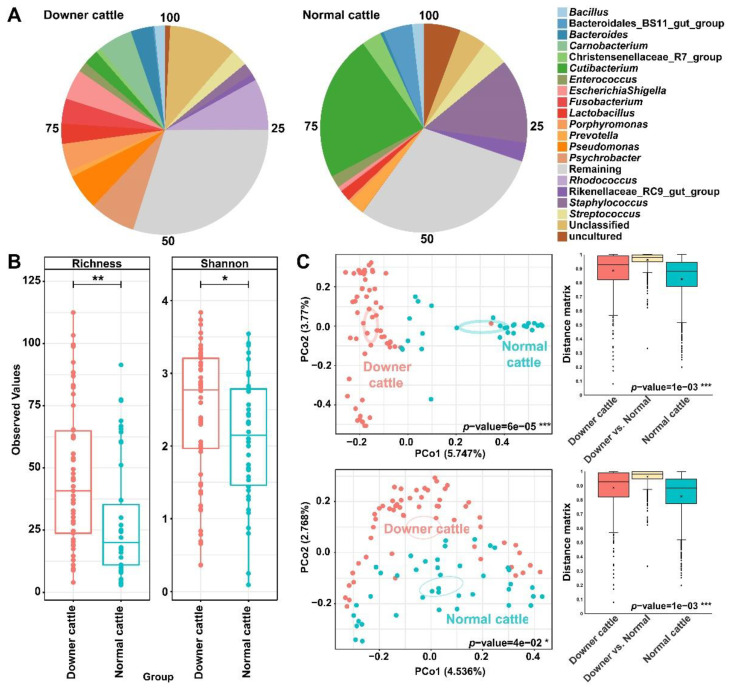
Relative abundance and microbial diversity of brain microbiota from downer and normal cattle. (**A**) Pie chart of relative abundance from downer and normal cattle’s microbiota at the genus level. (**B**) Alpha diversity indices (Shannon and Richness index) of downer and normal cattle groups based on the number of genera. (**C**) PCoA plot of beta diversity based on the Bray–Curtis (top) and Unweighted Unifrac (bottom) distance method and its distance matrix. Asterisks such as *, **, and *** mean *p*-values of 0.05, 0.01, and 0.001, respectively.

Before analyzing a microbiota, all samples were normalized as 3911 reads and it was confirmed that 3572 ASVs were obtained from the normalized reads. Variations of microbiota from three same brain tissue within samples were extremely low or not detected. The microbial community structure and diversity were compared between the downer and normal cattle. The bacterial community structure was illustrated based on the top 20 abundant genera (Figure 1A). *Rhodococcus*, *Psychrobacter*, *Pseudomonas*, *Carnobacterium*, *Escherichia*-*Shigella*, *Fusobacterium*, and *Porphyromonas* genera were more abundant in downer cattle. Furthermore, *Carnobacterium* genus was detected to some brain tissue samples from downer cattle in both results when bacteria isolation result from the diagnosis result and ASVs table in genus level were compared (Table S3). *Cutibacterium* and *Staphylococcus* genera were more abundant in normal cattle. Our results showed that opportunistic pathogenic bacteria were higher in downer cattle than in normal cattle. Shannon and Richness indices were calculated, and it was determined that two indices from downer cattle were significantly higher than normal cattle (Figure 1B). However, there is no significant difference in alpha diversity with an ASVs level when other metadata such as age, lesion, sex, kinds, and tissue were used (Figure S2). The distance between each sample was calculated according to Bray–Curtis dissimilarity and Unweighted UniFrac methods. In two principal coordinate analysis (PCoA) plots based on two distance calculation methods, sample differences in PCo1 and PCo2 were calculated as almost 5% and 3%, respectively. However, all samples that were classified into downer and normal cattle groups showed that the distance matrix values between the two were statistically different (*p* < 0.05, Figure 1C).

### 3.3. Representative Genera in the Brain Microbiota of the Downer and Normal Cattle

**Figure 2:**
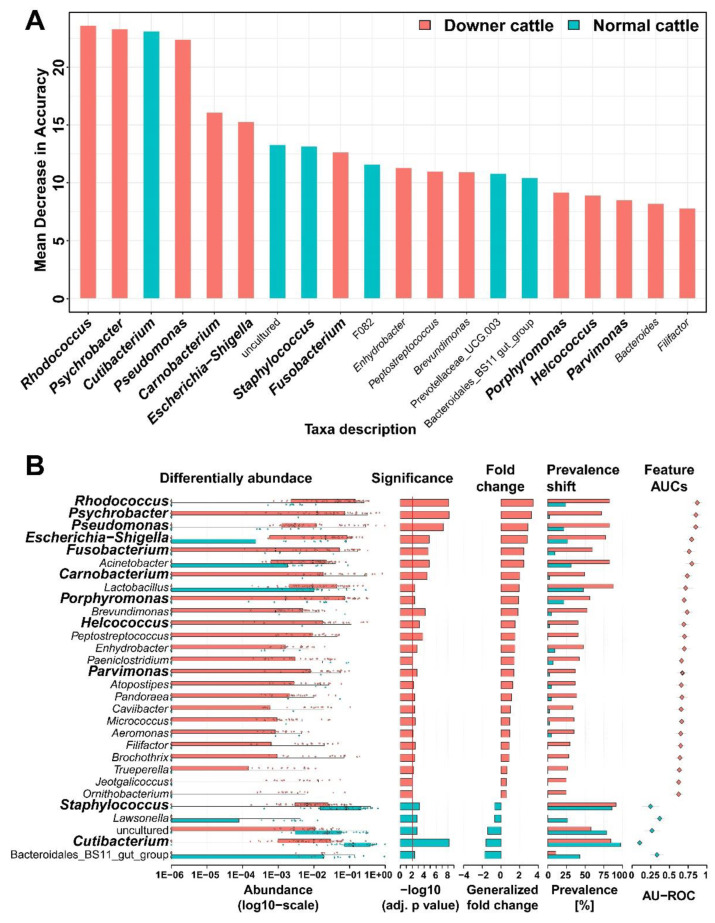
Investigation of the representative genus based on the downer and normal cattle’s microbiota. The genera written by bold character were detected in feature selection by Random Forest regression and a difference of their abundance in the microbial community at once. (**A**) Feature selection through Random Forest regression. (**B**) Association of the representative genus and its sample group through confirmation using association function in the SIAMCAT R package.

According to the random forest regression, feature selection at the genus level was performed to investigate the importance of abundant genera (Figure 2A). *Rhodococcus*, *Psychrobacter*, and *Pseudomonas* genus from downer cattle and *Cutibacterium* genus from normal cattle exhibit high mean decrease accuracy. Additionally, the selected genera were compared in terms of their differential abundance, significance, fold change, prevalence shift, and AUC score (Figure 2B). The genera in Figure 2B were significantly different between downer and normal cattle (*p* < 0.05). Additionally, genera, such as *Rhodococcus*, *Psycobacter*, *Pseudomonas*, *Escherichia*-*Shigella*, *Fusobacterium*, and *Carnobacterium* from downer cattle, and *Staphylococcus* and *Cutibacterium* from normal cattle exhibited high significance, fold change, and AUC scores. The AUC score, sensitivity, and specificity were calculated using the *Rhodococcus*, *Psychrobacter*, and *Pseudomonas* genera showing high differential abundance and fold change in Figure 2B. In the calculation result, the values such as AUC score, sensitivity, and specificity through three genera were higher than the values calculated using each (Figure S3). Therefore, it was considered that several genera highlighted in the results, such as *Rhodococcus*, *Psychrobacter*, and *Pseudomonas* may have the potential as etiological agent candidates for diagnosing DC.

### 3.4. Bacterial Network from Brain Microbiota between Downer and Normal Cattle

**Figure 3:**
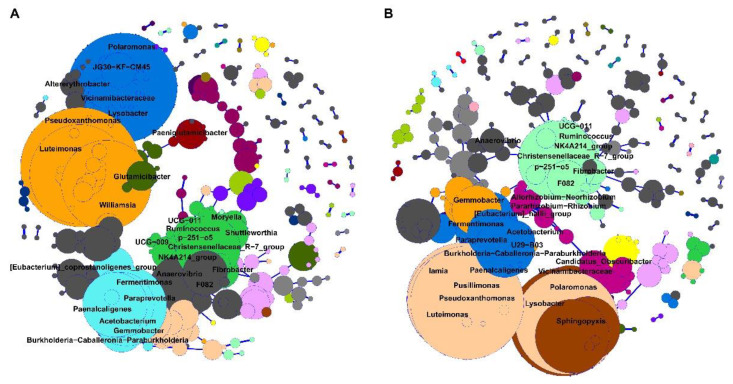
The bacterial network of brain microbiota. Bacterial networks were illustrated through genera with over 0.5 of *rho* value. In the network, the blue line indicates a positive correlation between the edges. The genus name is displayed on nodes with more than 5 edges. The node size and color corresponded to its total degree and sub-community, respectively. (**A**) Bacterial network for downer cattle. (**B**) Bacterial network for normal cattle.

Spearman’s correlation of downer and normal cattle’s bacterial networks was calculated and showed a positive correlation that is statistically significant (Figure 3). The numbers of nodes and edges from the downer cattle group were 213 and 531, respectively. In the normal cattle group case, the number of nodes and edges are 230 and 545, respectively. The microbial network did not change dramatically due to DC; however, there is a slight difference in the composition of the microbial network. The genus which has more than five edges with other genera was higher in the downer cattle’s bacterial networks than normal cattle. The genera *Polaromonas*, *Pseudoxanthomonas*, *Luteimonas*, Christensenellaceae_R-7, and *Paenalcaligenes* were detected in all groups, and the number of their degrees was over 10 (Table S4). These genera may play a role as hub genera in networks. Additionally, some genera were detected differently between groups. The genera, such as UCG-009, JG30-KF-CM45, *Altererythrobacter*, and *Moryella* were seen only in the downer cattle’s bacterial networks. In normal cattle cases, the genera, such as *Pusillimonas*, *Sphingopyxis*, *Iamia*, and *Candidatus*_*Obscuribacter* were detected (Table S5).

### 3.5. Predicted Functional genes in the Brain Microbiota between Downer and Normal Cattle

**Figure 4:**
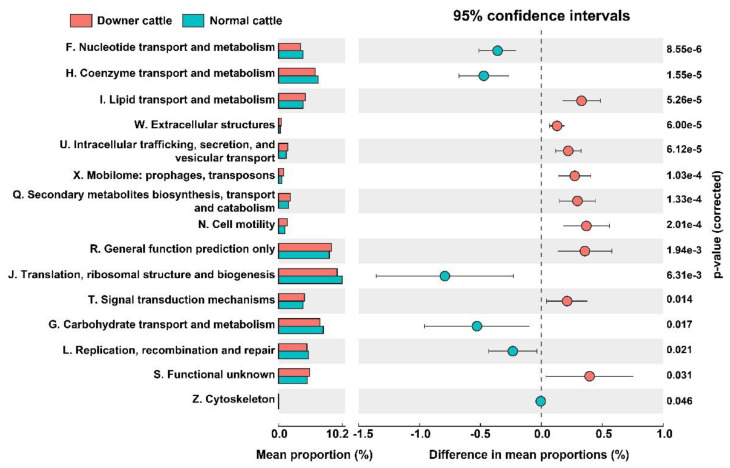
Functional gene prediction of brain microbiota from downer and normal cattle. The COG categories mentioned in the figure are statistically significant (*p* < 0.05). The mean portion was illustrated as a bar chart based on the detected COG IDs corresponding to its COG categories. The mean proportions, sphere, and error bar indicate mean and standard deviation, respectively. The corrected *p*-value was mentioned on the right.

To investigate the functional genes in the brain microbiota between downer and normal cattle, PICRUSt2 was used. 4472 clusters of orthologous (COD) IDs were detected and re-classified based on the COG categories. The differences in functional gene categories were statistically different (Figure 4). Gene groups higher in normal cattle were related to F. nucleotide transport and metabolism, H. coenzyme transport and metabolism, J. translation, ribosomal structure and biogenesis, G. carbohydrate transport and metabolism, and L. replication, recombination, and repair. However, COG categories, including N. cell motility, I. lipid transport and metabolism, extracellular structure, U. intracellular trafficking, secretion, vesicular transport, and Q. secondary metabolite biosynthesis, transport, and catabolism were significantly higher in downer cattle. Other genes in COG categories were not different (Table S6). At the pathway level, the number of functional genes in fatty acid biosynthesis was higher in downer cattle than in normal cattle. On the other hand, functional genes related to purine, pyrimidine, and thymidylate biosynthesis, glycolysis, heme and folate biosynthesis, and aminoacyl-tRNA synthetases were abundant in the normal cattle group (Table S7).

## 4. Discussion

Generally, downer cow (DC) can be caused by physiological factors such as mineral disorder and musculoskeletal problems, but neurological diseases such as bovine sponge encephalopathy (BSE), encephalitis, meningoencephalitis, and meningitis can cause DC [30,31]. However, DC with unknown etiology still detected and its etiological agent not been observed. Previous analysis revealed that microorganisms, such as bacteria, fungi, and the virus could invade brain tissue [11,12]. Thereby, studies related to microorganisms or microbiota using brain tissue or CSF samples are currently ongoing [32]. From the point of views, we analyzed the microbiota of brain tissue from the downer and normal cattle to investigate bacterial etiological agents for DC. According to our results, depending on the presence of downer cow (DC), the microbiota of brain tissue from downer and normal cattle are clearly different. Especially, representative genera from downer cattle have several previous studies related to nerval diseases such as encephalitis and meningitis to humans, animals, and other organisms.

Several representative genera (*Psychrobacter*, *Pseudomonas*, *Shigella*, *Fusobacterium*, *Porphyromonas*, *Rhodococcus*, *Helcococcus*, and *Parvimonas*) in downer cattle were significantly higher than normal cattle. *Psychrobacter* genus was isolated from the CSF sample of pediatric patient suffer from the meningitis and confirmed by metagenomic sequencing [33]. *Pseudomonas*, *Escherichia*, and *Shigella* genus were isolated and detected as pathogenic bacteria for encephalitis, valvular endocarditis, and bacterial meningoencephalitis to human and several bird [34,35,36,37,38]. Encephalitis and meningitis were outbreak by *Fusobacterium*, *Porphyromonas*, and *Rhodococcus* genus which is detected as pathogenic bacteria by isolation and polymerase chain reaction (PCR) to human [39,40,41,42]. Especially, movement disorder through complication of encephalitis was detected to mice which inject with *Rhodococcus* genus [43]. *Carnobacterium*, *Helcococcus*, and *Parvimonas* genus were detected as pathogenic bacteria for neurological disorder such as encephalitis, valvular endocarditis, and meningoencephalitis by isolation and NGS technique method to fish, bovine, and human [44,45,46,47,48,49]. According to these previous studies, it could be suggested that *Carnobacterium* and *Parvimonas* genera may cause these diseases through an invasion by way of the nasal cavity and oral flora, respectively, which move to the brain tissue. On the other hand, the genera *Cutibacterium* and *Staphylococcus* were significantly higher in normal cattle group. These genera can cause skin disease and mastitis in animals [50,51]. But the *Cutibacterium* genus case, it has revealed a beneficial effect on bovine rumen [52]. Even if representative genera from normal cattle such as *Cutibacterium* and *Staphylococcus* were known as pathogenic bacteria, these might not be recognized as bacterial etiological agents for DC with unknown etiology in this study.

Functional gene categories in the downer group including Q. secondary metabolite biosynthesis, transport and catabolism, T. signal transduction mechanism, I. lipid transport and metabolism, U. intracellular trafficking, secretion, and vesicular transport categories, which are significantly higher than the normal cattle group. There are previous studies that fatty acid and concentration of β-hydroxybutyric acid, apolipoprotein which related to functional gene categories in downer cattle group can cause milk fever and DC [53,54,55]. In addition, genes which include bacterial intracellular trafficking pathways, are related to bacterial toxins, and maybe a potential cause for DC [56]. On the other hand, several pathways including purine and pyrimidine biosynthesis, glycolysis, and heme biosynthesis which recognized as a microbial central metabolic pathway were higher in normal cattle group. According to previous studies, microbial metabolic pathways and their metabolites may help to host’s nerve system or cause neurological disease like Alzheimer’s disease [57,58]. However, it is difficult to explain the possibility that the predicted functional genes of microorganisms are directly induced or related to DC addressed in this study. Although these genes were hard to explain disease outbreaks directly, it was considered that these results are still remarkable as the potential to influence disease and phenotypes in the host.

Up to now, one microbial species was only considered a bacterial etiological agent for certain animal diseases. But, sometimes, diagnosing a disease is hard if its pathogenic bacteria are not detected. Therefore, a diagnosis method based on NGS technology and bioinformatic analysis can be a way to help the investigation of a bacterial etiological agent. In our result, *Rhodococcus*, *Psychrobacter*, and *Pseudomonas* genera were detected as representative genera and showed high AUC scores. And *Carnobacterium* genus existed in both microbiota and isolation data. Therefore, there is the possibility that these genera can be recognized as new pathogenic bacterial candidate although there needs to be future studies done.

In this study, differences in brain microbiota from downer and normal cattle were investigated through comparisons of microbial diversity, representative genus, microbial network, and functional gene. Age, sex, presence of lesion, and detection of pathogenic bacteria and viruses may influence an outbreak of the disease, and these are used widely in microbiota analysis as metadata. However, in this study, experiments were carried out using dead bovine, and since the economic value of normal cattle is very large, it was very difficult to collect normal cattle, which are female, similar in age to downer cattle in the process of collecting samples of normal cattle. In addition, it is considered that policies related to animals in the research field can induce a bias in the number of samples between downer and normal cattle in the sample collection step. Even if Shannon index calculation using these metadata did not show a statistically significant difference, it is expected that more accurate results will be obtained if the age and sex between the downer and normal cattle are similar. Therefore, a long-term study will be necessary to solve this problem, and our follow-up study will be conducted on the relationship between the DC and the metadata, microorganisms, and functional genes that differ in downer and normal cattle.

## 5. Conclusions

Up to now, there have been lots of microbiota studies to investigate pathogenic bacteria or etiological candidates for many animal diseases, especially for reproductive disorders such as abortion and diarrhea. Still, microbiota studies about neurological symptom are infrequent in the livestock industry. To the best of our knowledge, this study is the first to deal with microbiota differences by focusing on the brain tissue of downer and normal cattle. In this study, we analyzed differences in microbiota from brain tissue of downer and normal cattle. Differences in microbial diversity, representative genera, bacterial network, and predicted functional genes between the downer and normal cattle imply the possibility of microbiota difference for the outbreak of DC. Additionally, from the results of this study, a representative genera of downer cattle can potentially be a bacterial etiological agent candidate in DC diagnosis, and a research approach can be used to investigate a microbial etiological agent related to neurological disease in animals. Furthermore, there is a possibility that a machine learning model with random forest or support vector machine algorithm using the microbiota dataset which is similar to our study can help to diagnose downer cow with unknown etiology. From this point of view, the microbiota set of brain tissue from downer and normal cattle in this study can be a primary result, and other microbiota data sets from additional studies should be accumulated for the accuracy of the machine learning model.

## Figures and Tables

**Table 1:** The characteristics of downer and normal cattle.

Variable	Downer Cattle	Normal Cattle
**Number of brain tissue sample** **(Mixed brain, forebrain, midbrain, cerebellum, hindbrain, brainstem)**	57 (10, 11, 11, 9, 10, 6)	42 (0, 8, 9, 9, 8, 8)
**Number of bovine type** **(Korean beef, beef cattle, dairy cattle)**	21 (12, 1, 8)	9 (9, 0, 0)
**Average age of bovine sample (months)**	52.2	29.1
**Sex (male, female)**	9, 48	42, 0
**Number of brain samples with pathogenic bacteria isolated**	9	0
**Number of brain samples with virus detected**	5	0

## Data Availability

The information as follows was stated about sequencing raw data availability. Sequencing raw data in this study were deposited into the Sequence Read Achieve (SRA) of the National Center for Biotechnology Information (NCBI) (SRA accession number PRJNA634437).

## Supplementary Materials

The following supporting information can be downloaded at: https://www.mdpi.com/article/10.3390/microorganisms11010098/s1. Figure S1. The result of cross-contamination test to brain tissue samples. In the gel electrophoresis image, P means positive control (*Escherichia coli* DH5α), and N means negative control (sterilized distilled water). (A) 16S rRNA gene amplification result of DNA extraction solution and extracted DNA. (B) Amplicon sequencing library result using total DNA from the brain tissues. (C) Incubation result of third (70% ethanol) and final wastes (distilled water) on the 1/10× BHI agar plate. Figure S2. Shannon index calculation using various metadata in ASV level. (A) Shannon index toward age. (B) Shannon index toward the presence of a lesion. (C) Shannon index toward sex. (D) Shannon index toward kinds of cattle. (E) Shannon index toward brain tissue. Figure S3. Calculation of AUC score, sensitivity, and specificity by the number of cases. (A) ROC curves based on the calculation result. (B) Calculation results from the top three genera and their combination. Table S1. The list of bovine brain tissues based on the disease diagnostics in this study. In table O and X means the presence and absence of each brain tissue, respectively. Mixed brain sample indicates that all brain tissues were mixed. Table S2. Diagnosis results of downer and normal cattle. Table S3. The comparison of isolated bacteria and microbiota. Table S4. The list of genera that are shared in the bacterial network from downer and normal cattle. Table S5. The list of genera that are different in the bacterial network from downer and normal cattle. Table S6. Differences of COG categories from downer and normal cattle. The bold character in COG category means that it showed statistically significant. Table S7. Differences of COG pathway from downer and normal cattle.
