# Supplementary material for: Bacterial Profiles of Brain in Downer Cattle with Unknown Etiology
Source: Microorganisms. 2022 Dec 30;11(1):98. doi: 10.3390/microorganisms11010098 (PMC9862898; doi:10.3390/microorganisms11010098)
Supplement: Supplementary file 1 [file microorganisms-11-00098-s001.zip › Supplemental Table S1.pdf]

Table S1:
The list of bovine brain tissues based on the disease diagnostics in this study. In table O and X means the presence and absence of each brain tissue, respectively. Mixed brain sample indicates that all brain tissues were mixed.

Disease diagnostics	Sample name	Brain tissue (n = 57)				
		Forebrain	Midbrain	Hindbrain	Cerebellum	Brain stem
Downer cattle	19B38	O	O	O	O	X
	19B39	O	O	O	O	O
	19B40	O	O	O	O	O
	19B41	O	O	O	O	X
	19B42	O	O	O	O	X
	19B01	Mixed brain sample				
	19B02	O	O	X	X	X
	19B03	Mixed brain sample				
	19B12	Mixed brain sample				
	19B14	Mixed brain sample				
	19B23	Mixed brain sample				
	19B28	Mixed brain sample				
	19B29	Mixed brain sample				
	19B13	Mixed brain sample				
	19B15	Mixed brain sample				
	19B22	Mixed brain sample				
	19B33	O	O	O	O	O
	19B34	O	O	O	O	O
	19B35	O	O	O	O	O
	19B36	O	O	O	O	O
	19B37	O	O	O	X	X

Table S1. (Continued).

Disease diagnostics	Sample name	Brain tissue (n= 42)				
		Forebrain	Midbrain	Hindbrain	Cerebellum	Brain stem
Normal cattle	19R149	O	O	O	O	O
	19R150	O	O	O	O	O
	19R1601	O	O	O	O	O
	19R1603	O	O	O	O	O
	19R1604	O	O	O	O	O
	19R1651	O	O	O	O	O
	19R1652	O	O	O	O	O
	19R1653	X	O	X	O	O
	19R1656	O	O	O	O	X
