# Supplementary material for: Bacterial Profiles of Brain in Downer Cattle with Unknown Etiology
Source: Microorganisms. 2022 Dec 30;11(1):98. doi: 10.3390/microorganisms11010098 (PMC9862898; doi:10.3390/microorganisms11010098)
Supplement: Supplementary file 1 [file microorganisms-11-00098-s001.zip › Supplemental Table S2.pdf]

Table S2:
Diagnosis results of downer and normal cattle.

<table>
<thead>
<tr><th>Sample</th><th>Age (month)</th><th>Sex</th><th>Pathological diagnosis</th><th>Clinical symptom</th><th>Calcium conc. (mg/dL)</th><th>Magnesium conc. (mg/dL)</th><th>Pathogenic bacteria isolation</th><th>Virus detection</th><th>Antibody test</th><th>Post mortal change</th><th>Final diagnosis</th></tr>
</thead>
<tbody>
<tr><td>19B01.Brain</td><td>18</td><td>Male</td><td>Not detected</td><td>Lack of appetite, Intertia, High fever</td><td rowspan="10">within normal range (8.5-12.4mg/dL)</td><td rowspan="10">within normal range (1.8-3.2mg/dL)</td><td>No isolate</td><td>Not detected</td><td>Not detected</td><td>Not detected</td><td>Downer cattle</td></tr>
<tr><td>19B02.Forebrain</td><td>36</td><td>Female</td><td>Not detected</td><td>Not detected</td><td>No isolate</td><td>Not detected</td><td>Not detected</td><td>Not detected</td><td>Downer cattle</td></tr>
<tr><td>19B02.Midbrain</td><td>36</td><td>Female</td><td>Not detected</td><td>Not detected</td><td>No isolate</td><td>Not detected</td><td>Not detected</td><td>Not detected</td><td>Downer cattle</td></tr>
<tr><td>19B03.Brain</td><td>48</td><td>Female</td><td>Not detected</td><td>Sudden death</td><td>No isolate</td><td>Not detected</td><td>Not detected</td><td>Not detected</td><td>Downer cattle</td></tr>
<tr><td>19B12.Brain</td><td>49</td><td>Female</td><td>Not detected</td><td>Anxiety, Excitation, Ataxia, Unknown nonambulatory state</td><td>Carnobacterium divergens (brain)</td><td>Not detected</td><td>Not detected</td><td>Not detected</td><td>Downer cattle</td></tr>
<tr><td>19B13.Brain</td><td>36</td><td>Female</td><td>Mild non-suppurative encephalitis</td><td>Anxiety, Ataxia, Unknown nonambulatory state</td><td>No isolate</td><td>Not detected</td><td>Not detected</td><td>Not detected</td><td>Downer cattle</td></tr>
<tr><td>19B14.Brain</td><td>50</td><td>Female</td><td>Not detected</td><td>Depress, Ataxia, Unknown nonambulatory state</td><td>No isolate</td><td>Not detected</td><td>Not detected</td><td>Not detected</td><td>Downer cattle</td></tr>
<tr><td>19B15.Brain</td><td>16</td><td>Male</td><td>Moderate non-suppurative encephalitis</td><td>Depress, Ataxia, Unknown nonambulatory state, Unknown paralysis</td><td>Carnobacterium divergens (brain)</td><td>Not detected</td><td>Not detected</td><td>Not detected</td><td>Downer cattle</td></tr>
<tr><td>19B22.Brain</td><td>26</td><td>Female</td><td>Mild non-suppurative encephalitis</td><td>Depress, Ataxia, Unknown nonambulatory state, Unknown paralysis</td><td>No isolate</td><td>Not detected</td><td>Not detected</td><td>Not detected</td><td>Downer cattle</td></tr>
<tr><td>19B23.Brain</td><td>26</td><td>Female</td><td>Mild non-suppurative encephalitis</td><td>Depress, Ataxia, Unknown nonambulatory state, Unknown paralysis</td><td>No isolate</td><td>Not detected</td><td>Not detected</td><td>Not detected</td><td>Downer cattle</td></tr>
</tbody>
</table>

Table S2. (Continued)

Sample	Age (month)	Sex	Pathological diagnosis	Clinical symptom	Calcium conc. (mg/dL)	Magnesium conc. (mg/dL)	Pathogenic bacteria isolation	Virus detection	Antibody test	Post mortal change	Final diagnosis
19B28.Brain	62	Female	Not detected	Depress, Ataxia, Unknown nonambulatory state, Unknown paralysis	within normal range (8.5-12.4mg/dL)	within normal range (1.8-3.2mg/dL)	*Carnobacterium divergens* (brain)	Not detected	Not detected	Not detected	Downer cattle
19B29.Brain	75	Female	Not detected	Depress, Ataxia, Unknown nonambulatory state, Unknown paralysis			No isolate	Not detected	Not detected	Not detected	Downer cattle
19B33.Brainstem	27	Female	Mild lymphoblastic inflammation on the brain	Depress, Anxiety, Ataxia, Unknown nonambulatory state			No isolate	Not detected	Not detected	Not detected	Downer cattle
19B33.Cerebellum	27	Female	Mild lymphoblastic inflammation on the brain	Depress, Anxiety, Ataxia, Unknown nonambulatory state			No isolate	Not detected	Not detected	Not detected	Downer cattle
19B33.Forebrain	27	Female	Mild lymphoblastic inflammation on the brain	Depress, Anxiety, Ataxia, Unknown nonambulatory state			No isolate	Not detected	Not detected	Not detected	Downer cattle
19B33.Hindbrain	27	Female	Mild lymphoblastic inflammation on the brain	Depress, Anxiety, Ataxia, Unknown nonambulatory state			No isolate	Not detected	Not detected	Not detected	Downer cattle
19B33.Midbrain	27	Female	Mild lymphoblastic inflammation on the brain	Depress, Anxiety, Ataxia, Unknown nonambulatory state			No isolate	Not detected	Not detected	Not detected	Downer cattle
19B34.Brainstem	108	Female	Lymphocytic infiltration of cerebral blood vessel	Unknown nonambulatory state			No isolate	BVDV	Not detected	Not detected	Downer cattle
19B34.Cerebellum	108	Female	Lymphocytic infiltration of cerebral blood vessel	Unknown nonambulatory state			No isolate	BVDV	Not detected	Not detected	Downer cattle
19B34.Forebrain	108	Female	Lymphocytic infiltration of cerebral blood vessel	Unknown nonambulatory state			No isolate	BVDV	Not detected	Not detected	Downer cattle

Table S2. (Continued)

Sample	Age (month)	Sex	Pathological diagnosis	Clinical symptom	Calcium conc. (mg/dL)	Magnesium conc. (mg/dL)	Pathogenic bacteria isolation	Virus detection	Antibody test	Post mortal change	Final diagnosis
19B34.Hindbrain	108	Female	Lymphocytic infiltration of cerebral blood vessel	Unknown nonambulatory state	within normal range (8.5-12.4mg/dL)	within normal range (1.8-3.2mg/dL)	No isolate	BVDV	Not detected	Not detected	Downer cattle
19B34.Midbrain	108	Female	Lymphocytic infiltration of cerebral blood vessel	Unknown nonambulatory state			No isolate	BVDV	Not detected	Not detected	Downer cattle
19B35.Brainstem	74	Female	Severe suppurative encephalitis	Depress, Ataxia, Unknown nonambulatory state			*Trueperella pyogenes* (all)	Not detected	Not detected	Not detected	Downer cattle
19B35.Cerebellum	74	Female	Severe suppurative encephalitis	Depress, Ataxia, Unknown nonambulatory state			*Trueperella pyogenes* (all)	Not detected	Not detected	Not detected	Downer cattle
19B35.Forebrain	74	Female	Severe suppurative encephalitis	Depress, Ataxia, Unknown nonambulatory state			*Trueperella pyogenes* (all)	Not detected	Not detected	Not detected	Downer cattle
19B35.Hindbrain	74	Female	Severe suppurative encephalitis	Depress, Ataxia, Unknown nonambulatory state			*Trueperella pyogenes* (all)	Not detected	Not detected	Not detected	Downer cattle
19B35.Midbrain	74	Female	Severe suppurative encephalitis	Depress, Ataxia, Unknown nonambulatory state			*Trueperella pyogenes* (all)	Not detected	Not detected	Not detected	Downer cattle
19B36.Brainstem	38	Female	Non-suppurative encephalitis	Depress, Anxiety, Ataxia, Unknown nonambulatory state, Unknown paralysis			No isolate	Not detected	Not detected	Not detected	Downer cattle
19B36.Cerebellum	38	Female	Non-suppurative encephalitis	Depress, Anxiety, Ataxia, Unknown nonambulatory state, Unknown paralysis			No isolate	Not detected	Not detected	Not detected	Downer cattle
19B36.Forebrain	38	Female	Non-suppurative encephalitis	Depress, Anxiety, Ataxia, Unknown nonambulatory state, Unknown paralysis			No isolate	Not detected	Not detected	Not detected	Downer cattle

Table S2. (Continued)

Sample	Age (month)	Sex	Pathological diagnosis	Clinical symptom	Calcium conc. (mg/dL)	Magnesium conc. (mg/dL)	Pathogenic bacteria isolation	Virus detection	Antibody test	Post mortal change	Final diagnosis
19B36.Hindbrain	38	Female	Non-suppurative encephalitis	Depress, Anxiety, Ataxia, Unknown nonambulatory state, Unknown paralysis	within normal range (8.5-12.4mg/dL)	within normal range (1.8-3.2mg/dL)	No isolate	Not detected	Not detected	Not detected	Downer cattle
19B36.Midbrain	38	Female	Non-suppurative encephalitis	Depress, Anxiety, Ataxia, Unknown nonambulatory state, Unknown paralysis			No isolate	Not detected	Not detected	Not detected	Downer cattle
19B37.Forebrain	24	Male	Suppurative tonsillitis	Depress, Ataxia, Unknown nonambulatory state, Unknown paralysis			No isolate	Not detected	Not detected	Not detected	Downer cattle
19B37.Hindbrain	24	Male	Suppurative tonsillitis	Depress, Ataxia, Unknown nonambulatory state, Unknown paralysis			No isolate	Not detected	Not detected	Not detected	Downer cattle
19B37.Midbrain	24	Male	Suppurative tonsillitis	Depress, Ataxia, Unknown nonambulatory state, Unknown paralysis			No isolate	Not detected	Not detected	Not detected	Downer cattle
19B38.Cerebellum	32	Female	Not detected	Depress, Anxiety, Ataxia, Unknown nonambulatory state, Unknown paralysis			No isolate	Not detected	Not detected	Not detected	Downer cattle
19B38.Forebrain	32	Female	Not detected	Depress, Anxiety, Ataxia, Unknown nonambulatory state, Unknown paralysis			No isolate	Not detected	Not detected	Not detected	Downer cattle
19B38.Hindbrain	32	Female	Not detected	Depress, Anxiety, Ataxia, Unknown nonambulatory state, Unknown paralysis			No isolate	Not detected	Not detected	Not detected	Downer cattle
19B38.Midbrain	32	Female	Not detected	Depress, Anxiety, Ataxia, Unknown nonambulatory state, Unknown paralysis			No isolate	Not detected	Not detected	Not detected	Downer cattle
19B39.Brainstem	49	Female	Not detected	Depress, Ataxia, Unknown nonambulatory state, Unknown paralysis			No isolate	Not detected	Not detected	Not detected	Downer cattle

Table S2. (Continued)

Sample	Age (month)	Sex	Pathological diagnosis	Clinical symptom	Calcium conc. (mg/dL)	Magnesium conc. (mg/dL)	Pathogenic bacteria isolation	Virus detection	Antibody test	Post mortal change	Final diagnosis
19B39.Cerebellum	49	Female	Not detected	Depress, Ataxia, Unknown nonambulatory state, Unknown paralysis	within normal range (8.5-12.4mg/dL)	within normal range (1.8-3.2mg/dL)	No isolate	Not detected	Not detected	Not detected	Downer cattle
19B39.Forebrain	49	Female	Not detected	Depress, Ataxia, Unknown nonambulatory state, Unknown paralysis			*Carnobacterium maltaromaticum* (Forebrain)	Not detected	Not detected	Not detected	Downer cattle
19B39.Hindbrain	49	Female	Not detected	Depress, Ataxia, Unknown nonambulatory state, Unknown paralysis			No isolate	Not detected	Not detected	Not detected	Downer cattle
19B39.Midbrain	49	Female	Not detected	Depress, Ataxia, Unknown nonambulatory state, Unknown paralysis			*Carnobacterium maltaromaticum* (Midbrain)	Not detected	Not detected	Not detected	Downer cattle
19B40.Brainstem	72	Female	Not detected	Ataxia, Unknown nonambulatory state, Unknown paralysis			No isolate	Not detected	Not detected	Not detected	Downer cattle
19B40.Cerebellum	72	Female	Not detected	Ataxia, Unknown nonambulatory state, Unknown paralysis			No isolate	Not detected	Not detected	Not detected	Downer cattle
19B40.Forebrain	72	Female	Not detected	Ataxia, Unknown nonambulatory state, Unknown paralysis			No isolate	Not detected	Not detected	Not detected	Downer cattle
19B40.Hindbrain	72	Female	Not detected	Ataxia, Unknown nonambulatory state, Unknown paralysis			No isolate	Not detected	Not detected	Not detected	Downer cattle
19B40.Midbrain	72	Female	Not detected	Ataxia, Unknown nonambulatory state, Unknown paralysis			No isolate	Not detected	Not detected	Not detected	Downer cattle
19B41.Cerebellum	63	Male	Not detected	Unknown nonambulatory state, Pytalism, Neurological sign			*Carnobacterium maltaromaticum*, *Enterococcus faecalis* (Cerebellum, Hindbrain)	Not detected	Not detected	Not detected	Downer cattle

Table S2. (Continued)

Sample	Age (month)	Sex	Pathological diagnosis	Clinical symptom	Calcium conc. (mg/dL)	Magnesium conc. (mg/dL)	Pathogenic bacteria isolation	Virus detection	Antibody test	Post mortal change	Final diagnosis
19B41.Forebrain	63	Male	Not detected	Unknown nonambulatory state, Pytalism, Neurological sign	within normal range (8.5-12.4mg/dL)	within normal range (1.8-3.2mg/dL)	*Carnobacterium divergens*, *Enterococcus faecalis* (Forebrain, Midbrain)	Not detected	Not detected	Not detected	Downer cattle
19B41.Hindbrain	63	Male	Not detected	Unknown nonambulatory state, Pytalism, Neurological sign			*Carnobacterium maltaromaticum*, *Enterococcus faecalis* (Cerebellum, Hindbrain)	Not detected	Not detected	Not detected	Downer cattle
19B41.Midbrain	63	Male	Not detected	Unknown nonambulatory state, Pytalism, Neurological sign			*Carnobacterium divergens*, *Enterococcus faecalis* (Forebrain, Midbrain)	Not detected	Not detected	Not detected	Downer cattle
19B42.Cerebellum	57	Female	Not detected	Unknown nonambulatory state, Pytalism			No isolate	Not detected	Not detected	Not detected	Downer cattle
19B42.Forebrain	57	Female	Not detected	Unknown nonambulatory state, Pytalism			*Carnobacterium maltaromaticum*, *Enterococcus faecalis* (Forebrain)	Not detected	Not detected	Not detected	Downer cattle
19B42.Hindbrain	57	Female	Not detected	Unknown nonambulatory state, Pytalism			No isolate	Not detected	Not detected	Not detected	Downer cattle
19B42.Midbrain	57	Female	Not detected	Unknown nonambulatory state, Pytalism			*Carnobacterium divergens*, *Enterococcus faecalis* (Midbrain)	Not detected	Not detected	Not detected	Downer cattle
19R149.Brainstem	30	Male	Not detected	Not detected			No isolate	Not detected	Not detected	Not detected	Normal cattle
19R149.Cerebellum	30	Male	Not detected	Not detected			No isolate	Not detected	Not detected	Not detected	Normal cattle

Table S2. (Continued)

Sample	Age (month)	Sex	Pathological diagnosis	Clinical symptom	Calcium conc. (mg/dL)	Magnesium conc. (mg/dL)	Pathogenic bacteria isolation	Virus detection	Antibody test	Post mortal change	Final diagnosis
19R149.Forebrain	30	Male	Not detected	Not detected	within normal range (8.5-12.4mg/dL)	within normal range (1.8-3.2mg/dL)	No isolate	Not detected	Not detected	Not detected	Normal cattle
19R149.Hindbrain	30	Male	Not detected	Not detected			No isolate	Not detected	Not detected	Not detected	Normal cattle
19R149.Midbrain	30	Male	Not detected	Not detected			No isolate	Not detected	Not detected	Not detected	Normal cattle
19R150.Brainstem	29	Male	Not detected	Not detected			No isolate	Not detected	Not detected	Not detected	Normal cattle
19R150.Cerebellum	29	Male	Not detected	Not detected			No isolate	Not detected	Not detected	Not detected	Normal cattle
19R150.Forebrain	29	Male	Not detected	Not detected			No isolate	Not detected	Not detected	Not detected	Normal cattle
19R150.Hindbrain	29	Male	Not detected	Not detected			No isolate	Not detected	Not detected	Not detected	Normal cattle
19R150.Midbrain	29	Male	Not detected	Not detected			No isolate	Not detected	Not detected	Not detected	Normal cattle
19R1601.Brainstem	31	Male	Not detected	Not detected			No isolate	Not detected	Not detected	Not detected	Normal cattle
19R1601.Cerebellum	31	Male	Not detected	Not detected			No isolate	Not detected	Not detected	Not detected	Normal cattle
19R1601.Forebrain	31	Male	Not detected	Not detected			No isolate	Not detected	Not detected	Not detected	Normal cattle
19R1601.Hindbrain	31	Male	Not detected	Not detected			No isolate	Not detected	Not detected	Not detected	Normal cattle
19R1601.Midbrain	31	Male	Not detected	Not detected			No isolate	Not detected	Not detected	Not detected	Normal cattle
19R1603.Brainstem	30	Male	Not detected	Not detected			No isolate	Not detected	Not detected	Not detected	Normal cattle
19R1603.Cerebellum	30	Male	Not detected	Not detected			No isolate	Not detected	Not detected	Not detected	Normal cattle
19R1603.Forebrain	30	Male	Not detected	Not detected			No isolate	Not detected	Not detected	Not detected	Normal cattle

Table S2. (Continued)

Sample	Age (month)	Sex	Pathological diagnosis	Clinical symptom	Calcium conc. (mg/dL)	Magnesium conc. (mg/dL)	Pathogenic bacteria isolation	Virus detection	Antibody test	Post mortal change	Final diagnosis
19R1603.Hindbrain	30	Male	Not detected	Not detected			No isolate	Not detected	Not detected	Not detected	Normal cattle
19R1603.Midbrain	30	Male	Not detected	Not detected			No isolate	Not detected	Not detected	Not detected	Normal cattle
19R1604.Brainstem	29	Male	Not detected	Not detected			No isolate	Not detected	Not detected	Not detected	Normal cattle
19R1604.Cerebellum	29	Male	Not detected	Not detected			No isolate	Not detected	Not detected	Not detected	Normal cattle
19R1604.Forebrain	29	Male	Not detected	Not detected			No isolate	Not detected	Not detected	Not detected	Normal cattle
19R1604.Hindbrain	29	Male	Not detected	Not detected			No isolate	Not detected	Not detected	Not detected	Normal cattle
19R1604.Midbrain	29	Male	Not detected	Not detected			No isolate	Not detected	Not detected	Not detected	Normal cattle
19R1651.Brainstem	29	Male	Not detected	Not detected	within normal range (8.5-12.4mg/dL)	within normal range (1.8-3.2mg/dL)	No isolate	Not detected	Not detected	Not detected	Normal cattle
19R1651.Cerebellum	29	Male	Not detected	Not detected			No isolate	Not detected	Not detected	Not detected	Normal cattle
19R1651.Forebrain	29	Male	Not detected	Not detected			No isolate	Not detected	Not detected	Not detected	Normal cattle
19R1651.Hindbrain	29	Male	Not detected	Not detected			No isolate	Not detected	Not detected	Not detected	Normal cattle
19R1651.Midbrain	29	Male	Not detected	Not detected			No isolate	Not detected	Not detected	Not detected	Normal cattle
19R1652.Brainstem	28	Male	Not detected	Not detected			No isolate	Not detected	Not detected	Not detected	Normal cattle
19R1652.Cerebellum	28	Male	Not detected	Not detected			No isolate	Not detected	Not detected	Not detected	Normal cattle
19R1652.Forebrain	28	Male	Not detected	Not detected			No isolate	Not detected	Not detected	Not detected	Normal cattle
19R1652.Hindbrain	28	Male	Not detected	Not detected			No isolate	Not detected	Not detected	Not detected	Normal cattle

Table S2. (Continued)

Sample	Age (month)	Sex	Pathological diagnosis	Clinical symptom	Calcium conc. (mg/dL)	Magnesium conc. (mg/dL)	Pathogenic bacteria isolation	Virus detection	Antibody test	Post mortal change	Final diagnosis
19R1652.Midbrain	28	Male	Not detected	Not detected	within normal range (8.5-12.4mg/dL)	within normal range (1.8-3.2mg/dL)	No isolate	Not detected	Not detected	Not detected	Normal cattle
19R1653.Brainstem	28	Male	Not detected	Not detected			No isolate	Not detected	Not detected	Not detected	Normal cattle
19R1653.Cerebellum	28	Male	Not detected	Not detected			No isolate	Not detected	Not detected	Not detected	Normal cattle
19R1653.Midbrain	28	Male	Not detected	Not detected			No isolate	Not detected	Not detected	Not detected	Normal cattle
19R1656.Cerebellum	27	Male	Not detected	Not detected			No isolate	Not detected	Not detected	Not detected	Normal cattle
19R1656.Forebrain	27	Male	Not detected	Not detected			No isolate	Not detected	Not detected	Not detected	Normal cattle
19R1656.Hindbrain	27	Male	Not detected	Not detected			No isolate	Not detected	Not detected	Not detected	Normal cattle
19R1656.Midbrain	27	Male	Not detected	Not detected			No isolate	Not detected	Not detected	Not detected	Normal cattle
