# Supplementary material for: Bacterial Profiles of Brain in Downer Cattle with Unknown Etiology
Source: Microorganisms. 2022 Dec 30;11(1):98. doi: 10.3390/microorganisms11010098 (PMC9862898; doi:10.3390/microorganisms11010098)
Supplement: Supplementary file 1 [file microorganisms-11-00098-s001.zip › Supplemental Table S3.pdf]

Table S3:
The comparison of isolated bacteria and microbiota.

Symptom	Sample	Tissue	The isolated bacterium	Microbiota (Genus level)
**Downner cattle**	19B12	Cerebellum	*Carnobacterium divergens*	*Carnobacterium* (6.5%)
	19B15	Cerebellum	*Carnobacterium divergens*	*Carnobacterium* (59.4%)
	19B28	Cerebellum	*Carnobacterium divergens*	*Carnobacterium* (2.35%)
	19B35	Forebrain, Midbrain, Hindbrain, Cerebellum, Brainstem	*Trueperella pyogenes*	*Trueperell* (0.29%)
	19B39	Forebrain, Midbrain	*Carnobacterium maltaromaticum*	*Carnobacterium* (1.9%)
	19B41	Forebrain, Midbrain, Hindbrain, Cerebellum	*Carnobacterium divergens*<br>*Carnobacterium maltaromaticum*<br>*Enterococcus faecalis*	*Carnobacterium* (5.64%)<br>*Enterococcus* (7%)
	19B42	Forebrain, Midbrain	*Carnobacterium maltaromaticum*<br>*Enterococcus faecalis*<br>*Carnobacterium divergens*<br>*Lactococcus garvieae*	*Carnobacterium* (3.9%)<br>*Enterococcus* (3.0%)<br>*Lactococcus* (0.01%)
