# Supplementary material for: Bacterial Profiles of Brain in Downer Cattle with Unknown Etiology
Source: Microorganisms. 2022 Dec 30;11(1):98. doi: 10.3390/microorganisms11010098 (PMC9862898; doi:10.3390/microorganisms11010098)
Supplement: Supplementary file 1 [file microorganisms-11-00098-s001.zip › Supplemental Table S4.pdf]

Table S4:
The list of genera that are shared in the bacterial network from downer and normal cattle.

Genus	Degree	Downer cattle			Normal cattle		
		rho value	*p*-value	*q*-value	*rho* value	*p*-value	*q*-value
Polaromonas	21	0.75	1.9E-10	2.8E-10	0.64	3.5E-09	4.3E-09
Pseudoxanthomonas	20	0.57	8.6E-10	1.4E-09	0.52	5.3E-08	5.9E-08
Luteimonas	20	0.53	2.6E-08	2.9E-08	0.59	1.6E-10	3.1E-10
Christensenellaceae_R-7_group	11	0.59	1.0E-08	1.1E-08	0.58	1.5E-08	1.7E-08
Paenalcaligenes	10	0.72	6.9E-12	1.5E-11	0.73	6.2E-13	1.8E-12
Fermentimonas	9	0.69	8.4E-12	1.8E-11	0.68	2.1E-09	2.7E-09
Paraprevotella	8	0.79	6.2E-17	2.4E-16	0.71	6.9E-14	2.3E-13
NK4A214_group	8	0.63	1.8E-10	3.1E-10	0.59	9.0E-09	1.1E-08
Gemmobacter	8	0.65	1.7E-10	2.6E-10	0.69	1.3E-11	2.7E-11
Burkholderia-Caballeronia-Paraburkholderia	8	0.60	2.2E-10	4.0E-10	0.55	3.6E-09	5.4E-09
Vicinamibacteraceae	6	0.62	1.4E-11	3.4E-11	0.56	2.1E-09	3.3E-09
p-251-o5	6	0.62	7.3E-10	1.1E-09	0.65	2.7E-10	4.7E-10
F082	6	0.60	3.3E-09	4.4E-09	0.60	2.0E-08	2.1E-08
Ruminococcus	6	0.61	4.7E-09	5.5E-09	0.60	1.1E-08	1.2E-08
Lysobacter	6	0.62	7.5E-09	8.5E-09	0.62	2.5E-08	2.7E-08
UCG-011	5	0.67	9.1E-11	1.6E-10	0.68	5.0E-10	8.0E-10
Fibrobacter	5	0.63	3.0E-09	3.8E-09	0.65	1.4E-09	2.0E-09
Anaerovibrio	5	0.70	1.5E-11	3.2E-11	0.61	3.0E-11	6.7E-11
Acetobacterium	5	0.77	5.0E-11	9.1E-11	0.75	8.1E-10	1.2E-09
