# Supplementary material for: Bacterial Profiles of Brain in Downer Cattle with Unknown Etiology
Source: Microorganisms. 2022 Dec 30;11(1):98. doi: 10.3390/microorganisms11010098 (PMC9862898; doi:10.3390/microorganisms11010098)
Supplement: Supplementary file 1 [file microorganisms-11-00098-s001.zip › Supplemental Table S5.pdf]

Table S5:
The list of genera that are different in the bacterial network from downer and normal cattle.

Type	Genus	Degree	Average of *rho* value	Average of *p*-value	Average of *q*-value
Downer cattle	UCG-009	7	0.72	6.8E-12	1.5E-11
	JG30-KF-CM45	7	0.55	4.1E-09	5.7E-09
	Altererythrobacter	6	0.56	4.6E-09	6.3E-09
	Moryella	6	0.54	2.5E-08	2.8E-08
	Glutamicibacter	6	0.51	8.0E-08	8.5E-08
	Paraprevotella	5	0.65	1.1E-10	2.1E-10
	Williamsia	5	0.61	3.3E-08	3.4E-08
	Paeniglutamicibacter	5	0.60	6.9E-09	8.2E-09
	[Eubacterium]_coprostanoligenes_group	5	0.59	5.0E-08	5.1E-08
	Shuttleworthia	5	0.53	7.2E-08	7.4E-08
Normal cattle	*Pusillimonas*	21	0.52	5.0E-08	5.5E-08
	Sphingopyxis	19	0.60	1.3E-09	1.7E-09
	Iamia	16	0.57	1.3E-08	1.7E-08
	Candidatus_Obscuribacter	6	0.63	1.3E-08	1.5E-08
	U29-B03	6	0.53	2.6E-08	3.1E-08
	Allorhizobium-Neorhizobium-Pararhizobium-Rhizobium	5	0.80	1.4E-08	1.5E-08
	Christensenellaceae_R-7_group	5	0.55	2.0E-08	2.4E-08
	[Eubacterium]_hallii_group	5	0.51	8.0E-08	8.4E-08
