# Supplementary material for: Bacterial Profiles of Brain in Downer Cattle with Unknown Etiology
Source: Microorganisms. 2022 Dec 30;11(1):98. doi: 10.3390/microorganisms11010098 (PMC9862898; doi:10.3390/microorganisms11010098)
Supplement: Supplementary file 1 [file microorganisms-11-00098-s001.zip › Supplemental Table S6.pdf]

Table S6:

Differences of COG categories from downer and normal cattle. The bold character in COG category means that it showed statistically significant.

COG category	Downer cattle		Normal cattle		No. of functional genes	*p*-values (corrected)
	Mean relative frequency (%)	Standard deviation (%)	Mean relative frequency (%)	Standard deviation (%)		
A. RNA processing and modification	0.02	0.01	0.02	0.01	1	0.67071
B. Chromatin structure and dynamics	0.02	0.01	0.02	0.01	0	0.36330
C. Energy production and conversion	6.04	0.45	5.91	0.44	135	0.16685
D. Cell cycle control, cell division, chromosome partitioning	1.34	0.14	1.39	0.15	33	0.09869
E. Amino acid transport and metabolism	9.30	0.65	9.41	0.56	163	0.38351
**F. Nucleotide transport and metabolism**	**3.53**	**0.43**	**3.89**	**0.32**	**75**	**0.00001**
**G. Carbohydrate transport and metabolism**	**6.62**	**1.02**	**7.15**	**1.08**	**126**	**0.01660**
**H. Coenzyme transport and metabolism**	**5.86**	**0.48**	**6.33**	**0.52**	**116**	**0.00002**
**I. Lipid transport and metabolism**	**4.27**	**0.43**	**3.94**	**0.34**	**70**	**0.00005**
**J. Translation, ribosomal structure and biogenesis**	**9.37**	**1.59**	**10.16**	**1.20**	**164**	**0.00631**
K. Transcription	6.52	0.62	6.51	0.53	90	0.92842
**L. Replication, recombination and repair**	**4.54**	**0.60**	**4.77**	**0.37**	**91**	**0.02058**
M. Cell wall/membrane/envelope biogenesis	6.29	0.78	6.52	1.08	112	0.24342
**N. Cell motility**	**1.39**	**0.55**	**1.02**	**0.39**	**45**	**0.00020**
O. Posttranslational modification, protein turnover, chaperones	4.20	0.32	4.29	0.31	89	0.17398
P. Inorganic ion transport and metabolism	5.84	0.40	5.81	0.66	140	0.76377

Table S6. (Continued)

COG category	Downer cattle		Normal cattle		No. of functional genes	*p*-values (corrected)
	Mean relative frequency (%)	Standard deviation (%)	Mean relative frequency (%)	Standard deviation (%)		
**Q. Secondary metabolites biosynthesis, transport and catabolism**	**1.88**	**0.46**	**1.59**	**0.27**	**64**	**0.00013**
**R. General function prediction only**	**8.48**	**0.56**	**8.12**	**0.54**	**232**	**0.00194**
**S. Function unknown**	**4.98**	**0.78**	**4.59**	**0.95**	**431**	**0.03139**
**T. Signal transduction mechanisms**	**4.14**	**0.46**	**3.93**	**0.37**	**105**	**0.01393**
**U. Intracellular trafficking, secretion, and vesicular transport**	**1.46**	**0.33**	**1.24**	**0.18**	**62**	**0.00006**
V. Defense mechanisms	2.65	0.38	2.53	0.32	11	0.11145
**W. Extracellular structures**	**0.41**	**0.18**	**0.28**	**0.12**	**20**	**0.00006**
**X. Mobilome: prophages, transposons**	**0.83**	**0.29**	**0.56**	**0.35**	**72**	**0.00010**
**Z. Cytoskeleton**	**0.01**	**0.01**	**0.01**	**0.01**	**1**	**0.04584**
