# Supplementary material for: Bacterial Profiles of Brain in Downer Cattle with Unknown Etiology
Source: Microorganisms. 2022 Dec 30;11(1):98. doi: 10.3390/microorganisms11010098 (PMC9862898; doi:10.3390/microorganisms11010098)
Supplement: Supplementary file 1 [file microorganisms-11-00098-s001.zip › Supplemental Table S7.pdf]

Table S7:
Differences of COG pathway from downer and normal cattle.

Category	Pathway	Downer cattle		Normal cattle		No. of functional genes	p-values (corrected)
		Mean relative frequency (%)	Standard deviation (%)	Mean relative frequency (%)	Standard deviation (%)		
F. Nucleotide transport and metabolism	Purine biosynthesis	0.04	0.01	0.05	0.01	15	0.00143
	Pyrimidine biosynthesis	0.05	0.01	0.06	0.01	8	0.00235
	Pyrimidine salvage	0.04	0.02	0.05	0.02	8	0.01213
	Thymidylate biosynthesis	0.03	0.01	0.04	0.01	5	0.00816
	Arginine biosynthesis	0.05	0.01	0.06	0.01	1	0.04708
G. Carbohydrate transport and metabolism	Glycolysis	0.04	0.01	0.05	0.01	12	0.00510
	Pentose phosphate pathway	0.05	0.02	0.07	0.02	4	0.00564
	Gluconeogenesis	0.04	0.02	0.05	0.02	2	0.00819
	Isoprenoid biosynthesis	0.02	0.01	0.03	0.02	1	0.00600
H. Coenzyme transport and metabolism	Heme biosynthesis	0.03	0.01	0.04	0.01	10	0.00433
	Folate biosynthesis	0.04	0.01	0.05	0.01	7	0.01457
	Menaquinone biosynthesis	0.02	0.01	0.03	0.01	5	0.00277
	Pantothenate CoA biosynthesis	0.04	0.01	0.05	0.01	5	0.00110
	Thiamine biosynthesis	0.05	0.01	0.06	0.02	3	0.00095
	Lipoate biosynthesis	0.04	0.01	0.05	0.03	2	0.00255
	Aromatic amino acid biosynthesis	0.04	0.01	0.06	0.01	1	0.00000
	Cysteine biosynthesis	0.04	0.02	0.05	0.03	1	0.02184

Table S7. (Continued)

Category	Pathway	Downer cattle		Normal cattle		No. of functional genes	p-values (corrected)
		Mean relative frequency (%)	Standard deviation (%)	Mean relative frequency (%)	Standard deviation (%)		
J. Translation, ribosomal structure and biogenesis	Aminoacyl-tRNA synthetases	0.05	0.01	0.06	0.01	21	0.00324
	Translation factors	0.05	0.01	0.06	0.01	10	0.00811
	16S rRNA modification	0.06	0.01	0.07	0.01	7	0.00166
K. Transcription	RNA polymerase	0.05	0.01	0.06	0.01	3	0.00062
I. Lipid transport and metabolism	Fatty acid biosynthesis	0.10	0.03	0.07	0.03	10	0.00269
	Menaquinone biosynthesis	0.18	0.09	0.13	0.03	1	0.00041
	Non-phosphorylated Entner-Doudoroff pathway	0.07	0.04	0.03	0.03	1	0.00001
Q. Secondary metabolites biosynthesis, transport and catabolism	Biotin biosynthesis	0.04	0.03	0.02	0.02	1	0.00481
R. General function prediction only	Non-phosphorylated Entner-Doudoroff pathway	0.09	0.04	0.08	0.04	3	0.00109
	Pyrimidine degradation	0.12	0.07	0.09	0.05	1	0.00412
	Pyrimidine salvage	0.07	0.02	0.05	0.02	1	0.00012
