# Supplementary figures and images for: Bacterial Profiles of Brain in Downer Cattle with Unknown Etiology
Source: Microorganisms. 2022 Dec 30;11(1):98. doi: 10.3390/microorganisms11010098 (PMC9862898; doi:10.3390/microorganisms11010098)

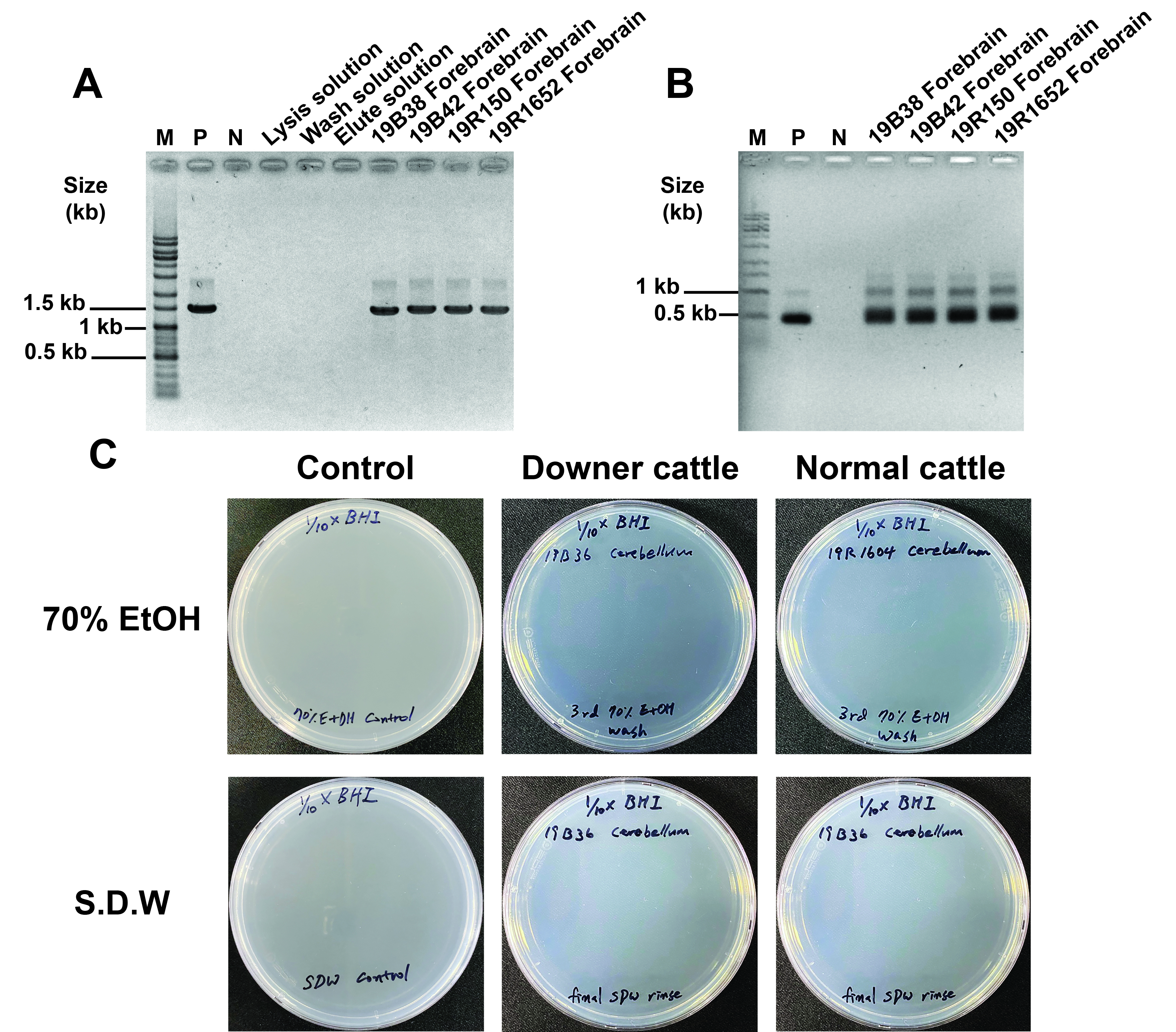

Supplement: Supplementary file 1 [file microorganisms-11-00098-s001.zip › supplemental Figure S1.jpg]

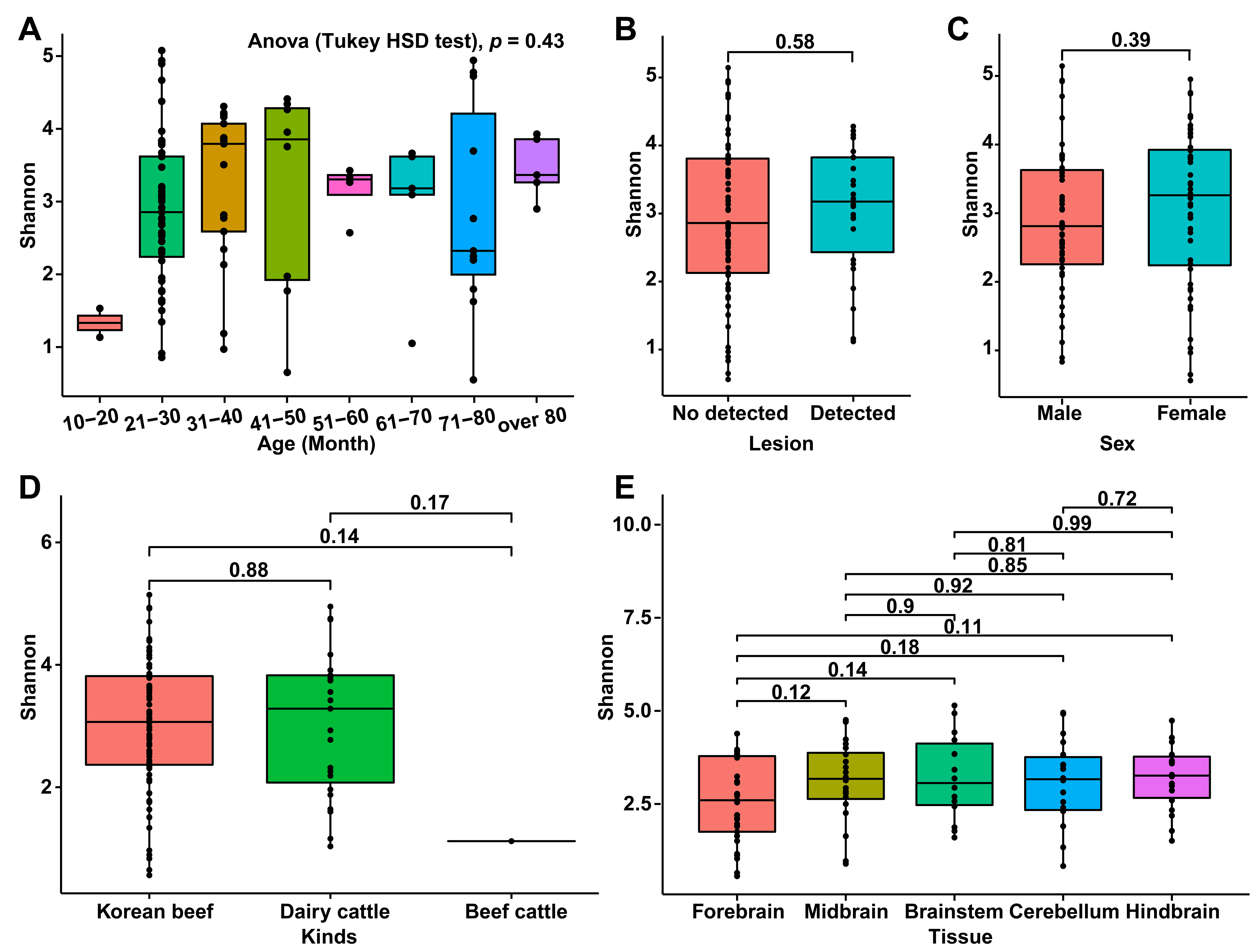

Supplement: Supplementary file 1 [file microorganisms-11-00098-s001.zip › supplemental Figure S2.jpg]

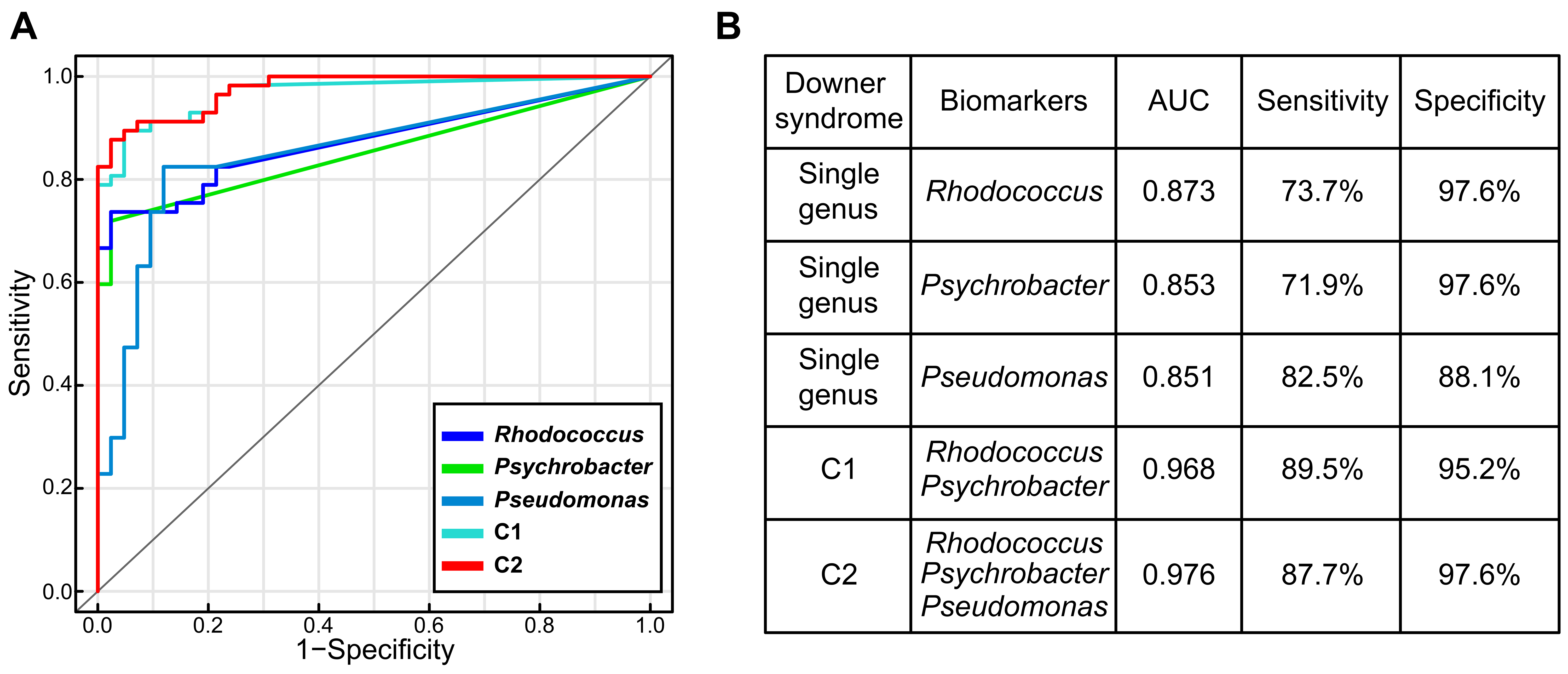

Supplement: Supplementary file 1 [file microorganisms-11-00098-s001.zip › supplemental Figure S3.jpg]
